# Bispecific antibodies targeting immunomodulatory checkpoints for cancer therapy

**DOI:** 10.20892/j.issn.2095-3941.2023.0002

**Published:** 2023-03-24

**Authors:** Tiancheng Zhang, Youpei Lin, Qiang Gao

**Affiliations:** 1 Department of Liver Surgery and Transplantation, and Key Laboratory of Carcinogenesis and Cancer Invasion (Ministry of Education), Liver Cancer Institute, Zhongshan Hospital, Fudan University, Shanghai 200032, China; 2 Key Laboratory of Medical Epigenetics and Metabolism, Institutes of Biomedical Sciences, Fudan University, Shanghai 200032, China; 3 State Key Laboratory of Genetic Engineering, Fudan University, Shanghai 200433, China

**Keywords:** Antibody–drug conjugate, bispecific antibody, immunotherapy, tumor microenvironment, clinical trials

## Abstract

Advances in antibody engineering have led to the generation of more innovative antibody drugs, such as bispecific antibodies (bsAbs). Following the success associated with blinatumomab, bsAbs have attracted enormous interest in the field of cancer immunotherapy. By specifically targeting two different antigens, bsAbs reduce the distance between tumor and immune cells, thereby enhancing tumor killing directly. There are several mechanisms of action upon which bsAbs have been exploited. Accumulating experience on checkpoint-based therapy has promoted the clinical transformation of bsAbs targeting immunomodulatory checkpoints. Cadonilimab (PD-1 × CTLA-4) is the first approved bsAb targeting dual inhibitory checkpoints, which confirms the feasibility of bsAbs in immunotherapy. In this review we analyzed the mechanisms by which bsAbs targeting immunomodulatory checkpoints and their emerging applications in cancer immunotherapy.

## Introduction

Antibodies are protective proteins produced by plasma cells that specifically bind to antigens. Due to the specific antigen binding ability, antibodies are widely used in scientific research, and some antibodies have been successfully transformed into clinical treatment. To date, a total of 131 antibodies have been approved for treatment or are under regulatory review by the U.S. Food and Drug Administration (FDA)^1^. Nearly one-half of the antibody-based treatments are used for cancer therapy, which mostly target programmed cell death-1 (PD-1), CD20, and human epidermal growth factor receptor 2 (HER2)^1^. The first monoclonal antibody (mAb) for use in immunotherapy was approved in 2011 targeting cytotoxic T lymphocyte antigen-4 (CTLA-4) in melanoma^2^. Subsequent development of immune-checkpoint inhibitors (ICIs) has revolutionized the standard treatment for patients with cancer; however, only a few patients who receive ICI show a durable response in clinical practice, demonstrating that the single mAbs have limited efficacy. Indeed, some combination therapies improve the response rate, but also exacerbate the side effects^3^. As the technology in antibody engineering continues to evolve, bispecific antibodies (bsAbs) have been designed that bind to two different epitopes, and have brought new optimism for cancer therapy.

The idea of fusing multiple specificities into one antibody can be traced back to the 1960s^4^. Subsequently, the concept of bispecific constructs was demonstrated by engineering bispecific molecules and two different rabbit cell types were bridged via a single bsAb^5^. When applied in immunotherapy, catumaxomab (CD3 × EpCAM), the first approved bsAb for cancer treatment, bridges tumor cells and T cells, representing a milestone in bsAb therapy^6^. Since then, emerging bsAbs have demonstrated great potential in cancer therapy. For example, blinatumomab (CD3 × CD19) has shown strong anti-tumor activity and acceptable toxicity for patients with B-precursor acute lymphoblastic leukemia^7^. In addition to engaging immune cells, bsAbs also have a role in payload delivery, co-factor mimicry, and receptor inhibition or activation^8^.

A balance between inhibitory and co-stimulatory receptors contributes to immune homeostasis under physiologic conditions^9^; however, such a balance is broken in the tumor microenvironment (TME), where inhibitory signaling is enhanced and co-stimulatory signaling is repressed. Blocking PD-1/PD-L1 signaling is the most successful immunotherapeutic strategy and has been included in the standard treatment for multiple cancers^10^. Pre-clinical and clinical data have shown that developing novel bsAbs targeting immune checkpoints is a promising approach to further improve the therapeutic effect. Herein we summarize the emerging checkpoint-targeted bsAbs and outline their potential clinical application.

## BsAb classification

**Figure 1:**
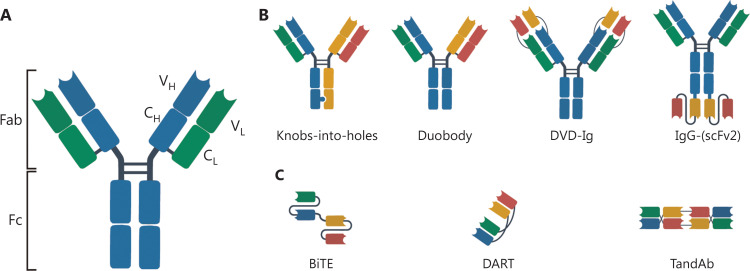
The immunoglobulin G (IgG) structure and schematic diagram of several representative bsAbs. The IgG is roughly “Y-shaped”. The two heavy chains are shown in blue and the two light chains are shown in green (A). IgG-like bsAbs (B). Non-IgG-like bsAbs (C). BiTE, bispecific T-cell engager; DART, dual-affinity retargeting molecule; DVD-Ig, dual-variable-domain immunoglobulin; scFv, single-chain variable fragment; TandAb, tandem diabody.

A natural antibody is roughly Y-shaped and consists of paired heavy (H) and light (L) chains. The antibody can be cleaved into two functional fragments by papain to yield Fab and Fc fragments (**Figure 1A**). The Fab fragment is the antigen-binding position, while the Fc fragment interacts with effector molecules and cells, thus participating in antibody-dependent cell-mediated cytotoxicity (ADCC), complement-dependent cytotoxicity (CDC), and antibody-dependent cellular phagocytosis (ADCP)^11^. Natural antibodies are often monospecific and bivalent with two identical antigen-binding sites. In contrast, bsAbs can only be generated by biochemical or genetic technologies and bind two distinct antigens. Conjugating two kinds of antibodies or fragments is the most common method used for producing bsAbs^5^. In addition, bsAbs can also be generated by hybrid hybridomas, which circumvents protein degradation during chain separation^12^; however, chain association restricts the productivity and subsequent clinical application of hybridoma technology^13^. The recent development of the species-restricted light chain pairing method has enhanced productivity of bsAbs by promoting the use of hybridoma technology. Based on the presence or absence of the Fc region, bsAbs can be divided into IgG-like and non-IgG-like bsAbs (**Figure 1B, 1C**).

### IgG-like bsAbs

IgG-like bsAbs are Y-shaped, in which the Fc fragment confers several advantages in manufacturing and clinical therapeutics. An intact antibody structure not only facilitates the purification process, but also increases the stability of the product^14^. In addition, the IgG-like bsAbs have a longer half-life *in vivo* compared to non-IgG-like bsAbs due to the neonatal Fc receptor (FcRn)-mediated recycling process. More importantly, the Fc fragment mediates the innate and adaptive immune responses, playing a crucial role in anti-tumor activity; however, there is a need for a more stringent quality control process due to chain association. IgG-like bsAbs mainly include knobs-into-holes, Duobody, IgG-(scFv2), and DVD-Ig^15^ (**Figure 1B**).

### Non-IgG-like bsAbs

Due to the lack of an Fc fragment, non-IgG-like bsAbs have unique advantages. The simple structure of the non-IgG-like bsAbs can be easily produced in eukaryotic and prokaryotic expression systems^16^. In addition, it is feasible to design the number of antigen-binding sites so that the valency of the two targets can be tailored. Non-IgG-like bsAbs mainly include BiTE, DART, and TandAb^17–19^ (**Figure 1C**).

## BsAbs reprogram the TME

The TME contains abundant immunosuppressive cells and ligands that dramatically dampen the efficacy of various anticancer immunotherapies. The anti-PD-1/PD-L1 mAbs relieve T cell dysfunction and reverse exhausted T cells^20^. Yet, anti-PD-1/PD-L1 therapy has several limitations. First, cancer and immunosuppressive cells in the TME express alternative inhibitory checkpoints beyond PD-L1, which leads to incomplete blockage of immunosuppressive signaling^9^. Second, anti-PD-1/PD-L1 therapy depends on the pre-existing immune response, but has limited effects on T cell priming and proliferation^21^. Thus, there is considerable interest in strategies that enhance the activation of effector T cells, such as co-stimulatory agonists and adoptive T cell therapy. Third, treatment with ICIs is accompanied by a high rate of immune-related adverse events (irAEs)^22^, which is mainly ascribed to the imbalance of inhibitory and co-stimulatory signaling in normal tissues^22^. Compared to mAbs, bsAbs can overcome these challenges. In this chapter we will discuss how bsAbs achieve a better therapeutic effect with a lower frequency of irAEs.

### Relieving the immunosuppressive phenotype

Inhibitory checkpoints and cytokines contribute to an immunosuppressive TME. In addition to the PD-1/PD-L1 axis, several inhibitory checkpoints have been identified and therapeutically validated, including CTLA-4, LAG-3, TIM-3, and TIGIT^9^. Compared to a combination of two mAbs, bsAbs bind to one cell that simultaneously expresses two antigens (in-cis binding) or two distinct cells that express one antigen (in-trans binding). In contrast, resistance is a major limitation of immunotherapy and often involves the upregulation of other inhibitory checkpoints, thus forming the basis for the exploitation of bsAbs that inhibit multiple checkpoints in an in-cis binding fashion. Most of these bsAbs are a combination of PD-1 and other checkpoint inhibitors. Previous data have shown that PD-1 × CTLA-4 and PD-1 × LAG-3 bsAbs have great potential for clinical application^23–25^. The in-trans binding reduces the distance and increases the contact between immune and cancer cells, thereby enhancing potential anti-tumor activity. Specifically, KN046, a PD-L1 × CTLA-4 bsAb, promotes the migration of T cells to the tumor site and the clearance of regulatory T cells (Tregs)^26^.

Pro-tumor growth factors (GFs) and cytokines modulate the immunosuppressive TME in multiple ways^27^. The composition of different cytokines in the TME determines the differentiation and function of immune cells. TGF-β, for example, stimulates the differentiation of naïve T cells into Treg cells independent of IL-6. Treg cells secrete TGF-β and IL-10, which further interferes with anti-tumor immunity. Several bsAbs targeting checkpoints and GFs/cytokines, such as YM101 (PD-L1 × TGF-β) and AK112 (PD-1 × VEGF), are under development. Therefore, bsAbs may efficiently circumvent immune tolerance in the TME.

### Enhancing the T cell-mediated immune response

Multiple approaches have been exploited to enhance T cell-mediated immunity, which includes cancer vaccines, adoptive T cell therapy, and immune agonist antibodies^21^. Co-stimulatory signaling pathways are vital to T cell priming, proliferation, differentiation, and effector function^20^. There are several mAbs targeting co-stimulatory molecules, such as 4-1BB, ICOS, and OX40, which are undergoing evaluation in clinical trials as single agents or in combination with ICIs^28^; however, there is no definitive evidence supporting the clinical benefit in patients receiving immune agonists in a large clinical trial.

Accumulating evidence has demonstrated that the bsAbs targeting co-stimulatory molecules have potent anti-tumor activity with low off-targeted toxicity^29,30^. Two bsAb designs have been shown to activate co-stimulatory signaling. One design depends on the engagement of tumor-associated antigens (TAAs), which simultaneously trigger the co-stimulatory signaling pathway and the recognition of tumor cells. IgG-like or non-IgG-like bsAbs, which crosslink 4-1BB and TAA, have shown robust anti-tumor activity and alleviation of toxicities^31,32^. The other design involves the integration of co-stimulatory and inhibitory checkpoints into the bispecific structure, which relieves the exhausted phenotype of the effector cells and overcomes the drug resistance associated with ICI therapy. The existing evidence has demonstrated that the exhaustion of T cells induces resistance to immune agonist monotherapy, which provides the rationale for the development of bsAbs^33^.

### Reducing immune-related adverse events

The clinical use of ICIs is confronted by many challenges, which include irAEs and drug resistance. Compared with PD-1 inhibitors, irAEs are more frequent and severe in patients receiving CTLA-4 inhibitors^22^. The occurrence of irAEs shows that immune checkpoint blockade can also activate an immune response in normal tissues. Numerous studies have been conducted to uncouple responses from toxicity.

A unique advantage of the bispecific structure is the in-trans binding of two types of cells or the in-cis binding of two molecules on the cell membrane. By adjusting the affinity of two binding sites, bsAbs reduce the off-target effect in normal tissues, making it possible to use drugs, the clinical application of which is restricted because of severe side effects. CD47, for example, can interact with membrane proteins of myeloid cells and activate “don’t-eat-me” signaling^34^. Indeed, malignant cells overexpress CD47 to evade the attack of the innate immune system in many cancers. Although CD47 antibodies have achieved a promising therapeutic effect in hematologic malignancies, the use of CD47 mAbs elicits severe anemia in patients with solid tumors^35^. After fusing CD47 and PD-L1 into a bsAb, there is preferential inhibition of CD47 in tumor and myeloid cells that express PD-L1, which significantly reduces the occurrence of anemia^36^.

## Specificities of bsAbs targeting immunomodulatory checkpoints

**Figure 2:**
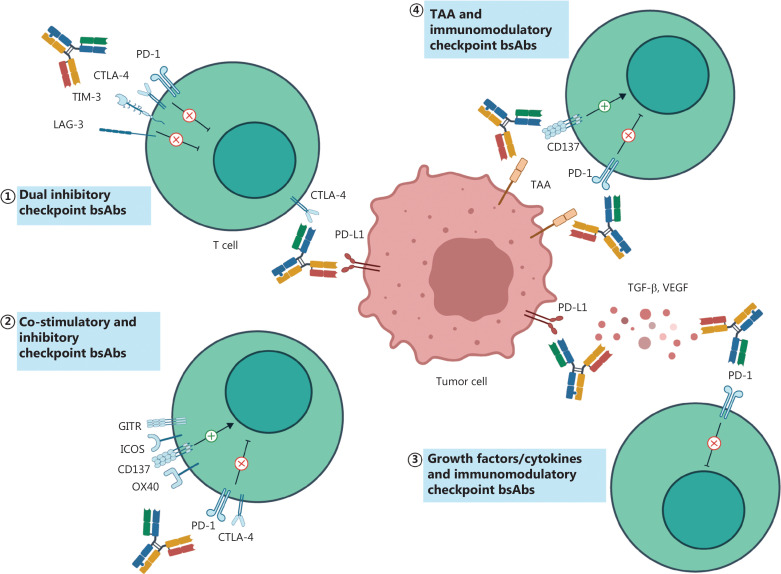
The bsAbs targeting immunomodulatory checkpoints. The checkpoint-targeted bsAbs are mainly divided into three categories: targeting dual inhibitory checkpoints (①); targeting co-stimulatory and inhibitory checkpoints (②); and targeting immunomodulatory checkpoints and non-checkpoint targets (③④). TAA, tumor-associated antigen; PD-L1, programmed death-ligand 1; TGF-β, transforming growth factor-β.

Immune checkpoints are classified into two types: co-stimulatory and inhibitory. Most checkpoint molecules belong to the immunoglobulin superfamily (IgSF, e.g., LAG-3) and tumor necrosis factor receptor superfamily (TNFRSF, e.g., 4-1BB)^9^. Abnormal expression of inhibitory checkpoints on cancer cells underlies immune evasion and drug resistance (**Figure 2**).

### Targeting dual inhibitory checkpoints

Since the approval of antibodies against CTLA-4 in 2011, ICIs have gradually become a standard treatment for various types of cancers^10^. Furthermore, a previous clinical study showed that ipilimumab is effective in improving the therapeutic response with pembrolizumab or nivolumab in patients with advanced renal cell carcinoma (RCC), advanced melanoma, and advanced non-small cell lung cancer (NSCLC)^10,37^; however, a combination of PD-1 and CTLA-4 inhibitors significantly increases the incidence of severe irAEs^22^. Surprisingly, preclinical and clinical findings demonstrated that PD-1 × CTLA-4 bsAbs have stronger anti-tumor activity compared to combination therapy, with manageable irAEs^38,39^. XmAb20717, which targets PD-1 and CTLA-4, has a higher affinity to PD-1/CTLA-4 dual-positive cells; the pre-clinical and clinical results have demonstrated tolerable side effects^40^. MEDI5752 is a novel monovalent bsAb that inhibits PD-1 and CTLA-4 signaling pathways, and preferentially binds to PD-1^+^ T cells in the TME^39^. Interestingly, the study also demonstrated that although the valence affects CTLA-4 inhibition, valence has little effect on PD-1^39^. A phase II dose-escalation and dose-expansion study indicated that MEDI5752 has promising antitumor activity in patients with advanced RCC^41^. Notably, among the bsAbs that target dual inhibitory checkpoints, cadonilimab was the first approved bsAb for patients with relapsed or metastatic cervical cancer^25^.

T cell immunoglobulin and mucin-domain containing 3 (TIM-3) regulates the immune response against tumors and leads to effector T cell dysfunction^42,43^. Upregulated expression of TIM-3 may mediate anti-PD-1 resistance, which can be reversed by a TIM-3 antibody^44^. RG7769, a heterodimeric TIM-3 × PD-1 bsAb with a high affinity to PD-1 and a low affinity to TIM-3, specifically binds to PD-1^+^ T cells and PD-1^+^ TIM-3^+^ T cells. Preclinical findings indicate that RG7769 promotes IFN-γ secretion from tumor-specific T cells and enhances anti-tumor activity of the tumor-infiltrating T cells^45^.

Lymphocyte activation gene-3 (LAG-3) was first reported as a molecule on activated T cells and a subset of NK cells in 1990, after which its negative regulation of cytotoxic T cells was discovered^46,47^. Moreover, because LAG-3 expression is closely related to PD-1 expression, simultaneously targeting LAG-3 and PD-1 is a promising treatment strategy^48–50^. The co-expression of LAG-3 and PD-1 has been shown to signify exhausted effector T cells in ovarian cancer, and thus simultaneous blocking of LAG-3 and PD-1 improves CD8^+^ T cell cytotoxicity^50^. Indeed, several agents targeting LAG-3 are under clinical investigation for various cancer types. Recently, the FDA approved relatlimab, a LAG-3 blocking antibody, in combination with nivolumab for unresectable or metastatic melanoma based on positive results from the RELATIVITY-047 study^51^. MGD013, a bispecific DART protein, has a high affinity for PD-1 and LAG-3. Compared with individual blockade of PD-1 or LAG-3 signaling, MGD013 demonstrates a stronger capacity for T cell activation and cytokine secretion^52^. In syngeneic mouse models of colorectal cancer (CRC), FS118 enhances the antitumor immune response and decreases the expression of LAG-3 on T cells, which may be due to shedding of LAG-3 from the cell surface to serum^24,53,54^. Dendritic cells (DCs) can process and present TAAs, which are crucial in initiating the anti-tumor immune response. ABL501, a LAG-3 × PD-L1 bsAb, induces the maturation of DCs by blocking PD-L1 signaling, thus augments CD8^+^ T cell cytotoxicity^55^. In a humanized mouse model, ABL501 has a dose-dependent anti-tumor response through the reinvigoration of immune cells^56^.

T cell immunoglobulin and ITIM domain (TIGIT) is an inhibitory checkpoint belonging to IgSF and is expressed on effector T, memory T, NK, and Treg cells. PVR (CD115) and PVRL2 (CD112) bind to the TIGIT and are expressed on tumor, T, and antigen-presenting cells (APCs). TIGIT competes with co-stimulatory receptors (CD226 and CD69), thereby interfering with the activation of immune cells. Recently, tiragolumab, an anti-TIGIT antibody, in combination with atezolizumab, has been approved as a breakthrough therapy for NSCLC patients with high PD-L1 expression^57^. This encouraging result suggests that TIGIT is a potential target and can synergize with other ICIs. A novel bsAb targeting TIGIT and PD-L1 has also been reported, in which the bsAb promotes IL-2 secretion from T cells *in vitro* and improves the overall survival in a transgenic mouse model^58^. PVR-related immunoglobulin domain-containing (PVRIG) is another inhibitory molecule that binds to PVRL2, and its inhibitory ability is partially independent of the TIGIT/PVR/PVRL2 axis^59^. A fully-human bsAb co-binding TIGIT and PVRIG shows robust anti-tumor activity *in vitro* and *in vivo*^60^.

In summary, the immunology of inhibitory checkpoints is a cornerstone of bsAb exploitation. In addition to the aforementioned checkpoints, new checkpoint molecules are being discovered on a regular basis. Due to the undefined functions, some of the checkpoint molecules, such as B7-H5 (VISTA), NKG2A, and BTLA, have not been considered to be bsAb targets^61^; however, the function of parental antibodies cannot effectively predict the combination of targets. Therefore, performing high throughput functional screening may guide the selection of potent bsAbs in the near future.

### Simultaneously targeting co-stimulatory and inhibitory checkpoints

Given that the prerequisite for the efficacy of ICIs is the pre-existing immune response, a combination of co-stimulatory agonists and checkpoint inhibitors may have a synergistic effect in maximizing the therapeutic response. Numerous biomedical companies have conducted clinical trials in this field (NCT02132754, NCT02553499, and NCT02598960).

As a member of the TNFRSF, glucocorticoid-induced TNF receptor (GITR) is a co-stimulatory checkpoint which mediates T cell activation and compromises immunosuppression of Treg cells^62^. To date, several anti-GITR mAbs have been generated. MEDI1873, a novel GITR agonist, shows great tolerance and pharmacodynamic activity, albeit lacking a promising clinical response^63^. Interestingly, anti-GITR antibody regulates tumor-infiltrating T cells and decreases the expression of TIGIT in combination with anti-PD-1 mAb^64^. This study provides the basis for the exploration of the bispecific structure targeting GITR and other immune checkpoints. To overcome resistance, a multimeric GITR ligand is fused with an anti-PD-1 mAb to form a bispecific construct. This bsAb triggers the infiltration of T cells, but reduces Treg and exhausted T cells^65^. Except for PD-1, another inhibitory checkpoint (CTLA-4) is under consideration as a potential bsAb target. ATOR114, a GITR×CTLA-4 bsAb, not only enhances the activation of T and NK cells, but also induces the depletion of Treg cells as well as GITR^+^ tumor cells^66^.

Notably, 4-1BB, also referred to as CD137 or TNFRSF9, has a key role in T cell proliferation and effector function^67^. A previous study showed that 4-1BB escalated the clinical efficacy of second- or third-generation chimeric antigen receptors (CARs) as a co-stimulatory receptor^68^. To date, the safety and therapeutic efficacy of 4-1BB agonists (urelumab and utolimumab) have been investigated in clinical trials. Unfortunately, limited efficacy with severe hepatotoxicity was obseved^69,70^. The bispecific construct may provide a resolution in reducing the toxicity of 4-1BB agonist by restricting its stimulatory capacity in specific immune cells. A novel 4-1BB × PD-L1 bsAb, ABL503, exhibits good tolerance by specifically stimulating intra-tumoral T cells^71^. Data from preclinical experiments and a phase I study showed that GEN1046, another bsAb targeting 4-1BB and PD-L1, regresses tumor growth by increasing the proliferation of T cells and cytokine secretion, including IFN-γ, IL-10, and CXCL10^72,73^.

OX40 is an inducible co-stimulatory checkpoint that is transiently expressed on CD4^+^ and CD8^+^ T cells following T cell receptor activation. In contrast, the OX40 molecule is constitutively expressed on intra-tumoral Treg cells. OX40 signaling does not elicit depletion of Treg cells and does not interfere with the immunosuppressive capacity of Treg cells, which is different from GITR^74^. In mouse models of ovarian cancer, PD-1 blockade plus OX40 stimulation has a synergistic effect in enhancing the infiltration of effector immune cells and inducing a tumor-specific T cell response, demonstrating the feasibility of combining OX40 with other checkpoints^75^. Two PD-L1 single-domain antibodies and an anti-OX40 antibody have been combined to generate a bsAb. The OX40 × PD-L1 bsAb exerts its stimulatory function and exhibits a dose-dependent anti-tumor response in various tumor mouse models^76^. With a different design strategy, the bsAb targeting OX40 and CTLA-4 reduces the density of Treg cellos in the TME due to the CTLA-4 moiety and activates effector and memory T cells^77^.

ICOS is a member of the IgSF and confers a stimulatory signal. Expression of ICOS depends on the activation of TCR and is constitutively expressed in Treg cells. Engagement of FcγR is essential in the mediation of different functions of antibodies, which includes agonism or induction of ADCC^78^. For example, vopratelimab is an anti-ICOS mAb and functions by reducing ICOS^+^ T cells, which are mostly Treg cells in the TME^79^. In contrast, GSK3359609, an anti-ICOS mAb, stimulates effector T cells but does not induce a decrease in the number of effector T cells^80^. KY105 is a novel ICOS × PD-L1 IgG1 bsAb that activates ICOS in a PD-L1-dependent manner. In a murine model of CRC, KY105 has demonstrated an encouraging anti-tumor efficacy by depleting ICOS^+^ Treg cells and increasing IFN-γ secretion^81^.

Collectively, fusing co-stimulatory and inhibitory checkpoints into a bispecific construct has two benefits. Specifically, bsAbs bridge tumor and immune cells, and simultaneously reinvigorate the proliferation and activities of T cells to improve clinical efficacy. In contrast, bsAbs reduce the side effects induced by co-stimulation signaling.

### Targeting immune checkpoints and non-checkpoint targets

#### Targeting immune checkpoints and TAAs

TAAs serve not only as a biomarker for the identification of tumor cells, but also as a target of novel cancer treatments, such as mAbs, cancer vaccines, and CAR-T therapy. ICI monotherapy lacks tumor selectivity, which results in the loss of specificity to tumor cells coupled with unpredictable adverse effects. Binding TAAs with immunomodulatory checkpoints presents a potential solution to these problems.

EpCAM is expressed in many normal epithelial tissues and is also recognized as a biomarker of cancer stem cells^82^. A CD40 × EpCAM bsAb has been designed to satisfy the need of tumor antigens in the activation of DC^83^. The bsAb promotes the priming of tumor-specific T cells and prolongs the overall survival in murine models^83^. Members of the ErbB family are receptor tyrosine kinases, including EGFR, HER2, HER3, and HER4, the aberrant activation of which induces tumor initiation and invasion^84^. Recently, a symmetric bsAb that blocks EGFR and PD-1 has been developed^85^. In addition to inhibition of EGFR signaling, the anti-PD1×EGFR bsAb triggers ADCC in tumor cells and leads to tumor shrinkage in xenografted and syngeneic CRC models^85^. In addition, two anti-PD-1 single-chain variable fragments are fused with anti-HER2 IgG to form a bsAb, which only blocks PD-1 signaling in the HER2-overexpressing TME^86^.

#### Targeting immune checkpoints and GFs/cytokines

GFs and cytokines have pro- and anti-tumor roles in tumor initiation and progression. As a result, there are many GFs and cytokine-targeted drugs that have been approved for clinical treatment as monotherapy or in combination with ICIs. For example, ICI plus anti-vascular endothelial growth factor (VEGF) antibody has become the first-line treatment option for patients with unresectable hepatocellular carcinoma (uHCC). Compared with GFs, cytokines constitute a more complex immunomodulatory network and have two-side effects on tumor immunity. It is important to recognize a specific context of the TME in which cytokines exert anti- rather than pro-tumor activities. Fusing GF or cytokines into immune checkpoint-targeted bsAbs not only enhances the efficacy of immunotherapy, but also precisely guides GF/cytokine therapy to the tumor region.

Beyond inhibition of angiogenesis, anti-VEGF therapy has immunostimulatory effects on immune cells in the TME. Previous clinical studies reported a superior patient response to the combined strategy of anti-VEGF and anti-PD-1/PD-L1 treatment in multiple cancer types^3^. AK112 is a first-in-class bsAb targeting VEGF and PD-1. Based on a successful experience of combined therapy, AK112 is under clinical assessment involving different types of cancer, and the preliminary results have been encouraging therapeutic responses^87,88^. TGF-β suppresses tumor growth in the early stage, but promotes drug resistance and metastasis in the advanced stage. Previous studies have demonstrated that hyperactivation of TGF-β signaling attenuates anti-PD-1/PD-L1 treatment, and dual blockade of TGF-β and PD-1/PD-L1 confers synergistic effects^89^. YM101, a novel bsAb targeting PD-L1 and TGF-β, acts on each step of the cancer-immunity cycle, which includes promoting antigen presentation, upregulating T cell infiltration, and enhancing tumor cell killing. YM101 shows stronger anti-tumor activity than single anti-TGF-β or anti-PD-L1 treatment *in vitro* and *in vivo*^90^.

Taken together, our analysis shows that there are two underlying mechanisms of bsAbs targeting immune checkpoints and non-checkpoint targets: (1) the specificity of TAAs functions as a “navigation system”, which exerts stimulatory or inhibitory effects on immune cells in the TME rather than in normal tissues; (2) the specificity of GFs/cytokines function as a “reinforcer”, which improves the tumor-killing effects and reduces drug resistance.

## Clinical trials assessing bsAbs targeting immunomodulatory checkpoints

### Inhibitory checkpoint × inhibitory checkpoint

Based on the encouraging data from a phase II study, cadonilimab is the first bsAb targeting dual inhibitory checkpoints which has been approved for marketing and used for the treatment of relapsed or metastatic cervical cancer. In a recent phase I/II clinical trial (NCT03852251), cadonilimab plus oxaliplatin and capecitabine showed good tolerance and a favorable objective response rate (ORR = 66.7%) as a first-line treatment option for advanced gastric or gastroesophageal junction cancer^91^. A phase II clinical trial (NCT04444167) evaluated the combination of cadonilimab and lenvatinib in patients with uHCC; an ORR of 44.4% was achieved with a manageable safety profile^92^. After the approval of cadonilimab, many types of clinical studies have been conducted involving cadonilimab (NCT05430906, NCT05377658, and NCT05227651). In addition, a phase I study involving vudalimab, another PD-1×CTLA-4 bsAb, showed vudalimab to be well-tolerated in ICI-pretreated patients with advanced cancer and an ORR of 13%^38^. In addition, a phase II study involving vudalimab as monotherapy or combined with chemotherapy or targeted agents in metastatic castration-resistant prostate cancer patients has been initiated^93^. A recent phase I trial also reported the efficacy and safety of MGD019 (NCT03761017) in advanced solid tumors, in which 4 of 25 patients had an objective response^94^.

RG7769 is a TIM-3×PD-1 bsAb developed by Roche^45^. A phase I study involving RG7769 (NCT03708328) was initiated to confirm the safety and tolerability in patients with solid tumors, but the study was discontinued^45^. A recent phase II study (NCT04785820) was initiated to compare RG7769 with nivolumab in advanced or metastatic esophageal squamous cell carcinoma. In addition, a phase Ia/b study of a TIM-3 × PD-L1 bsAb was terminated in the early stage of the study due to unexpected drug immunogenicity^95^.

Relatlimab, a first-in-class anti-LAG-3 antibody, was recently approved by the FDA to treat patients with melanoma in combination with nivolumab^96^. Among the bsAbs targeting LAG-3, tebotelimab is a DART bsAb that binds to LAG-3 and PD-1^97^. In a phase I clinical trial, advanced HER2^+^ neoplasm patients receiving tebotelimab plus margetuximab (an anti-HER2 mAb) had a preliminary ORR of 40% without unexpected side effects^98^. Another phase I study reported that tebotelimab has promising early evidence of anti-tumor activity in relapsed or refractory diffuse large B-cell lymphoma with acceptable patient tolerance^99^. FS118 is a novel bsAb fusing LAG-3 binding sites into an anti-PD-L1 IgG1 antibody^24,54^. A phase I study involving FS118, first-in-human, was conducted to assess the safety and activity in patients with advanced malignancies^100^. ABL501 is another investigational bsAb targeting LAG-3 and PD-L1; the preclinical data demonstrated a strong tumor response^55,56^. In an ongoing phase I study (NCT05101109), the maximum tolerated dose (MTD) of ABL501 was evaluated in unresectable or metastatic solid tumors. In addition, the MTD and recommended dose of XmAb22841, a LAG-3×CTLA-4 bsAb, in tandem with pembrolizumab are being evaluated in an ongoing DUET-4 trial (NCT03849469).

The CITYSCAPE phase II clinical trial (NCT03563716) investigated the possibility of tiragolumab, an anti-TIGIT mAb, plus atezolizumab for metastatic NSCLC. The data showed that the ORR was significantly prolonged in the combination group compared to the placebo plus atezolizumab group (31.3% *vs.* 11.2%, respectively; *P* = 0.05)^57^. Based on the positive result, the FDA granted approval for tiragolumab plus atezolizumab as a breakthrough therapy for NSCLC patients. Both HLX301 and PM1022 are bsAbs that bind to TIGIT and PD-L1^58,101^. The bsAbs targeting TIGIT are all under phase I clinical trials, albeit lacking clinical efficacy results. Ongoing clinical trials of bsAbs targeting dual inhibitory checkpoints are detailed in **Table 1**.

**Table 1:** Clinical trials of bsAbs targeting immunomodulatory checkpoints

Targets	Bispecific antibody	Combination agents	Condition	Phase	NCT number
Dual inhibitory checkpoints					
PD-1×CTLA-4	Cadonilimab (AK104)		Mesothelioma	I	NCT03261011
		Lenvatinib	uHCC	II	NCT04444167
		Cisplatin/carboplatin+ paclitaxel ± bevacizumab	Cervical cancer	III	NCT04982237
		Oxaliplatin + capecitabine	G/GEJ cancer	Ib/II	NCT03852251
	Vudalimab(XmAb20717)		Solid tumor	I	NCT03517488
			mCRPC	II	NCT05005728
		Carboplatin + cabazitaxel	mCRPC	II	NCT05005728
		Olaparib	mCRPC	II	NCT05005728
	MGD019		Solid tumor	I	NCT03761017
	MEDI5752		Solid tumor	I	NCT03530397
PD-L1×CTLA-4	KN046		Solid tumor	II	NCT04469725
TIM-3×PD-1	RG7769		Solid tumor	I	NCT03708328
			ESCC	II	NCT04785820
	AZD7789		Solid tumor	I/IIa	NCT04931654
TIM-3×PD-L1	LY3415244		Solid tumor	I	NCT03752177
LAG-3×PD-1	MGD013	Margetuximab	HER2+ neoplasms	I	NCT03219268
LAG-3×PD-L1	ABL501		Solid tumor	I	NCT05101109
	FS118		Solid tumor	I/II	NCT03440437
	IBI323		Advanced malignancies	I	NCT04916119
LAG-3×CTLA-4	XmAb22841	Pembrolizumab	Solid tumor	I	NCT03849469
TIGIT×PD-1	IBI321		Solid tumor	I	NCT04911881
TIGIT×PD-L1	HLX301		Solid tumor	I/II	NCT0510221
Co-stimulatory and inhibitory checkpoints					
4-1BB×PD-L1	INBRX-105	Pembrolizumab	Solid tumor	I	NCT03809624
	GEN1046		Solid tumor	I	NCT03917381
	ABL503		Solid tumor	I	NCT04762641
OX40×PD-L1	KN052		Solid tumor	I	NCT05309512
OX40×CTLA-4	ATOR-1015		Solid tumor	I	NCT03782467
ICOS×PD-1	XmAb23104	Ipilimumab	Solid tumor	I	NCT03752398
CD27×PD-L1	CDX-527		Solid tumor	I	NCT04440943
Immunomodulatory checkpoints and non-checkpoint targets					
4-1BB×Claudin 18.2	TJ-CD4B(ABL111)		Solid tumor	I	NCT04900818
VEGF×PD-1	AK112		Solid tumor	I	NCT04047290
			uHCC	II	NCT05432492
		Chemotherapy	SCLC	II	NCT05116007
		Chemotherapy	NSCLC	II	NCT04736823
			NSCLC	III	NCT05184712
		Chemotherapy	mCRC	II	NCT05382442
		Nab-paclitaxel/paclitaxel	TNBC	II	NCT05227664
		PARP inhibitor	rOC	I/II	NCT04999605
VEGF×PD-L1	HB0025		Solid tumor	I	NCT04678908

Source: http://www.clinicaltrials.gov (accessed on 13 November 2022). ESCC, esophageal squamous cell carcinoma; G/GEJ cancer, gastric/gastroesophageal junction cancer; mCRC, metastatic colorectal cancer; mCRPC, metastatic castration-resistant prostate cancer; NSCLC, non-small cell lung cancer; rOC, recurrent ovarian carcinoma; SCLC, small cell lung cancer; TNBC, triple-negative breast cancer; uHCC, unresectable hepatocellular carcinoma.

Most bsAbs that bind to dual inhibitory checkpoints are in the clinical development stages. Discovery of new inhibitory checkpoints and evaluation of their functions are crucial for further development of bsAbs.

### Co-stimulatory checkpoint × inhibitory checkpoint

Researchers have also investigated the clinical value of these co-stimulatory molecules. In 2006 a phase I study was conducted to test the safety of TGN1412, a CD28 agonist^102^. It was found that six volunteers had cytokine release syndrome and multiorgan failure^102^. This finding suggests that there are safety concerns regarding the use of co-stimulatory checkpoint agonists. Several clinical trials focusing on GITR, OX40, 4-1BB, and ICOS, which are safer relative to CD28, are ongoing.

ATOR-1144 is a tumor-directed bsAb that binds to GITR and CTLA-4^66^. Although ATOR-1144 has been shown to increase Treg depletion and activate the tumor response, the efficacy of ATOR-1144 has not been tested in clinical trials. INBRX-105 is a novel bsAb designed to activate 4-1BB signaling in regions with high PD-L1 expression. In an ongoing phase I study (NCT03809624), the safety profile of INBRX-105 alone or in combination with pembrolizumab in the treatment of solid tumors is under investigation. GEN1046 is an investigational 4-1BB × PD-L1 bsAb. A phase I/IIa study (NCT03917381) reported that GEN1046 administration resulted in disease control in 40/61 patients (65.6%) with various types of cancer^29^. In addition, several 4-1BB × PD-L1 bsAbs, including ATG-101 (NCT04986865), ABL503 (NCT04762641), and QLF31907 (NCT05150405), are currently being evaluated in phase I clinical trials to test the efficacy in patients with advanced solid tumors. OX40 plays an important role in T cell differentiation, activation, and maintenance of memory T cells^103^. A phase I clinical trial of ATOR-1015, a CTLA-4 × OX40 bispecific antibody, reported modest efficacy with an acceptable safety profile in advanced solid tumors^104^. Another phase I study involving KN052 targeting OX40 and PD-L1 in patients with advanced solid tumors in China is currently ongoing (NCT05309512). Feladilimab, a non-depleting IgG4 ICOS agonist antibody, has been evaluated in several clinical trials. In 2021, the phase II INDUCE-3 (NCT04128696) and phase II INDUCE-4 (NCT04428333) clinical trials were terminated, suggesting that the clinical efficacy of the ICOS agonist needs to be further explored. This finding partially reflects the difficulties of exploiting agents that target co-stimulatory checkpoints. A list of clinical trials mentioned above is given in **Table 1**.

In summary, the bsAbs targeting co-stimulatory and inhibitory checkpoints are all under evaluation in the early stage of clinical trials. A deeper understanding of how co-stimulatory signals work and their roles in the biology of T cell function will be helpful in developing this type of agent.

### Immunomodulatory checkpoints × non-checkpoint targets

The most studied TAAs (EpCAM, EGFR, and HER2) have been designed for specificity in bispecific molecules, but they are not yet in clinical trials. TJ-CD4B (ABL111) is the only bsAb that binds to 4-1BB and Claudin 18.2, and has been approved as an orphan drug for the treatment of gastric cancer^30^. A phase I clinical trial (NCT04328831) in patients with advanced malignancies showed that IBI322 (CD47 × PD-L1 bispecific antibody) is well-tolerated with a good tumor response (ORR = 20%)^105^. Apart from bsAbs that crosslink TAAs and checkpoints, several bsAbs targeting GFs/cytokines are now undergoing clinical trials. AK112 is an investigational humanized IgG1 VEGF × PD-1 bsAb. A phase I clinical trial (NCT04047290) evaluated the efficacy of AK112 in the treatment of patients with solid tumors. AK112 achieved an encouraging ORR of 23.5% with a satisfactory safety profile^87^. In the same phase I study, AK112 also exhibited potential clinical benefits [disease control rate (DCR) = 76.5%] in platinum-resistant/refractory epithelial ovarian cancer patients^88^. In a phase Ib/II trial (NCT04900363), AK112 exerted significant anti-tumor efficacy (ORR = 60%) as first- or second-line treatment for NSCLC^106^. AK112 plus chemotherapy showed encouraging clinical benefits in terms of improved progression-free survival (PFS) and ORR with manageable toxicity in NSCLC patients^107^. The bsAbs targeting immunomodulatory checkpoints and non-checkpoint targets are listed in **Table 1**.

In conclusion, targeting TAAs increases the accumulation of bsAb in tumor tissues, thereby achieving strong anti-tumor activity and restraining irAEs. Targeting GFs and cytokines may provide an additional immunotherapy strategy for cancer therapy; however, there are several important considerations for constructing this type of bsAb, including the selection of optimal TAAs and GFs/cytokines, determination of stimulatory or inhibitory checkpoints, and an appropriate bsAb backbone.

## Discussion

ICI therapy is a new type of cancer therapy that is increasingly being studied alone or in combination regimens. The novel biotechnology offers an opportunity to incorporate two antigen-binding sites into a single antibody. For agents that bind to immunomodulatory checkpoints, the advantages of the bispecific constructs include blocking two distinct inhibitory signaling, delivering agonistic signaling to specific immune cells in an immunosuppressive TME, and localizing stimulative or inhibitory effects in selected tumor tissues. In recent years, cadonilimab, the first approved bsAb targeting dual inhibitory checkpoints, was authorized by the National Medical Products Administration.

To achieve better clinical effects, numerous advanced technologies, including high-throughput target screening and antibody engineering, have been explored to discover new target combinations and the structure of antibodies. A recent study indicated that an unbiased functional screen of the bsAb library in patient-derived organoids can be used to select an optimal candidate^108^. Beyond the two specificities, multi-specific antibodies have been developed^109^. These results suggest a high requirement for understanding tumor immunology, checkpoint signaling, and protein structure.

Currently, the generation and application of bsAbs has three main challenges. The first challenge is that the fixed drug match and antibody valence limit adjustment of dose or therapeutic strategy. Second, the multi-signal activation may result in unknown immune effects, which warrants more exploration of the interaction between novel immune checkpoints. Third, bsAbs are designed with various formats to fulfill the required size, stability, and tissue penetration. The distinct formats may escalate the agent immunogenicity, thereby inducing anti-drug antibodies.

In summary, bsAbs targeting immunomodulatory checkpoints benefit from the successful experience of ICIs and advanced biotechnology of antibody generation. With the ongoing research in the mechanism of the bsAbs and the continuous optimization of bispecific molecule constructs, bsAbs are expected to be a novel agent for cancer therapy.
